# 

**Published:** 1903-09-05

**Authors:** 


### NOTICE TO CORRESPONDENTS.

All MSS., Letters, Books for Review, and other matters intended for the Editor should be addressed The Editor, "The Hospital," The Hospital Buildings, 28 & 29 Southampton Street, Strand, W.C.

The Editor cannot undertake to return rejected MS., even when accompanied by stamped directed envelope.

**Notice to Advertisers and Subscribers.**

All Advertisements, Orders for Copies of The Hospital, and Business Communications should be addressed to **THE MANAGER, (Not to the Editor)**, The Hospital, 28 & 29 Southampton Street, Strand, London, W.C.

The Cost of Subscription, post free, is as follows.—Per week, 3½d.; per quarter, 3s. 9d.; per half-year, 7s. 6d.; per annum, 15s. Foreign and Colonial:—Per annum, 19s. 6d., payable, in all cases, in advance.

**NOTICE.—Vol. XXXIII. of "The Hospital" (October 1902 to March 1903) in handsome cloth binding, will shortly be on sale at the office of this Journal, price 7s. 6d. Cases for binding the Numbers contained in this Volume 1s. 6d. Index to Vol. XXXIII., 2½d., post free.**

**The monthly part for September is now ready. Price 1s., by post 1s. 3d.**

### THE STUDENT OF MEDICINE.

### APPOINTMENTS.

**H. A. Easton**, M.R.C.S, L.R.C P, Medical Officer to the No. 4 District of Croydon Union. **Henry Charles Groves**, L.R.C.P. and S.I., Medical Officer of Health to the Monmouth Rural District Council. **Arthur King**, M.B., C M., D.P.H., R.C.P.I., re-appointed Medical Officer of Health to the Watford Urban District Council. **Sylvanus Glanville Morris**, M.D.Edin., Surgeon to the Lockets Merthyr Collieries, Mardy, Glam. **E. W. Rayment**, M.B., C.M, Medical Officer of Health to the Pewsey Rural District Council. **D. Ap. Simon**, M.B., Ch.B. District Medical Officer and Public Vaccinator for the Western District of Llandilofawr Union. **Henry Soltau**, L.R.C.P. and S.E., L.F.P.S.Glasg., Superintendent and Secretary of the Medical Missionary Association.

St. Thomas's Hospital.—The following gentlemen have been selected as House Officers from Tuesday, September 1st, 1903:—House Physicians: **P. N. Panton**, B.A.Cantab., L.R.C.P., M.R.C.S.; **J. N. Sargeant**, L.R.C.P., M.R.C.S.; **O. Hildesheim**, B.A., M.B., B.Ch.Oxon. (*Extension*); **H. W. Sexton**, L.R.C.P., M.R.C.S. (*Extension*). House Physicians to Out-patients: **R. E. H. Leach**, M.A., M.B., B.Ch.Oxon., L.R.C.P., M.R.C.S.; **G. R. Rickett**, M.A., B.C.Cantab. House Surgeons: **J. E. Adams**, L.R.C.P., M.R.C.S.; **H. Upcott**, L.R.C.P., M.R.C.S.; **C. Wheen**, B.A.Oxon., L.R.C.P., M.R.C.S.; **N. Carpmael**, L.R.C.P., M.R.C.S. House Surgeons to Out-patients: **W. L. Harnett**, B.A.Cantab., L.R.C.P., M.R.C.S.; **H. I. Pinches**, B.A., M.B., B.C.Cantab., L.R.C.P., M.R.C.S.; **T. Guthrie**, B.A., B.C.Cantab., L.R.C.P., M.R.C.S.; **L. E. Wigram**, M.A., M.B., B.C.Cantab. Obstetric House Physicians: (*Senior*) **H. Spurrier**, B.A.Cantab., L.R.C.P., M.R.C.S.; (*Junior*) **W. H. Harwood-Yarred**, B.Sc.Lond., L.R.C.P., M.R.C.S. Ophthalmic House Surgeon: (*Senior*) **A. C. Hudson**, M.A., M.B., B.C.Cantab. Clinical Assistants: (Throat) **J. Coates**, L.R.C.P., M.R.C.S. (*Extension*); **G. C. Adeney**, L.R.C.P., M.R.C.S. (Skin) **C. N. Sears**, L.R.C.P., M.R.C.S.; **C. H. Latham**, L.R.C.P., M.R.C.S. (Ear) **E. A. Ross**, M.B., B.C.Cantab. (*Extension*).

THE BEST NATURAL APERIENT WATER.

### Hunyadi János

For LIVER COMPLAINTS, OBESITY, &c.

The "VIENNA MEDICAL PRESS" says:—

"Hunyadi János may be regarded as a specific for obesity."

Average Dose.—*A wineglassful before breakfast, either pure or diluted with a similar quantity of hot or cold water.*

CAUTION.—Note the name "Hunyadi János," the signature of the Proprietor, ANDREAS SAXLEHNER, and the Medallion, on he Red Centre Part of the Label.

### Special Text-Books for Nurses.

### OPHTHALMIC NURSING.

By SYDNEY STEPHENSON, F.R.C.S.E.

Price **3/6** net, **3/10** post free.

A valuable guide on the Nursing of Eye Cases.

### NURSING IN DISEASES OF THE THROAT, NOSE, AND EAR.

By P. MACLEOD YEARSLEY, F.R.C.S.

Price **2/6.**

Contains useful hints on these special subjects.

### THE ART OF MASSAGE.

By A. CREIGHTON HALE.

Price **6/-.**

Every Nurse must find a knowledge of Massage useful and interesting.

### FEVERS AND INFECTIOUS DISEASES.

By Dr. WILLIAM HARDING.

Price **1/-.**

An excellent little handbook.

### NOTES ON PHARMACY AND DISPENSING.

By C. J. S. THOMPSON.

Price **1/-.**

An admirable introduction to Dispensing.

### MENTAL NURSING.

By Dr. WILLIAM HARDING.

Price **1/-.**

Full of useful advice to Nurses in Mental Cases.

### OUTLINES OF INSANITY.

By Dr. WALMSLEY.

A Treatise on the Salient Features of Insanity. Very useful to the intelligent Mental Nurse.

*Obtainable of all Booksellers, and published by*
THE SCIENTIFIC PRESS, LIMITED, 28 & 29 Southampton Street, Strand, London,
*who will send their latest Catalogue of Nursing Text-books post free to any Nurse on application.*

